# Lysosome-Associated Membrane Protein Targeting Strategy Improved Immunogenicity of Glycoprotein-Based DNA Vaccine for Marburg Virus

**DOI:** 10.3390/vaccines12091013

**Published:** 2024-09-04

**Authors:** Xiyang Zhang, Yubo Sun, Junqi Zhang, Hengzheng Wei, Jing Wang, Chenchen Hu, Yang Liu, Sirui Cai, Qinghong Yuan, Yueyue Wang, Yuanjie Sun, Shuya Yang, Dongbo Jiang, Kun Yang

**Affiliations:** 1 Department of Immunology, The Key Laboratory of Bio-Hazard Damage and Prevention Medicine, Basic Medicine School, Air Force Medical University (The Fourth Military Medical University), Xi’an 710032, China; xiyang72@fmmu.edu.cn (X.Z.); sunyubo000103@fmmu.edu.cn (Y.S.); zjq000316@fmmu.edu.cn (J.Z.); 15738168191@163.com (H.W.); wangjingfmmu@fmmu.edu.cn (J.W.); huchen111@fmmu.edu.cn (C.H.); caisirui@fmmu.edu.cn (S.C.); qinghongyuan@fmmu.edu.cn (Q.Y.); wangyyue66@fmmu.edu.cn (Y.W.); yuanjiesun@fmmu.edu.cn (Y.S.); yangsy@fmmu.edu.cn (S.Y.); 2 Military Medical Innovation Center, Air Force Medical University (The Fourth Military Medical University), Xi’an 710032, China; 3 Institute of AIDS Prevention and Control, Shaanxi Provincial Center for Disease Control and Prevention, Xi’an 710054, China; liuyangyang9610@163.com

**Keywords:** Marburg virus (MARV), DNA vaccine, lysosome-associated membrane protein (LAMP), Marburg hemorrhagic fever (MHF)

## Abstract

Marburg hemorrhagic fever (MHF) is a fatal infectious disease caused by Marburg virus (MARV) infection, and MARV has been identified as a priority pathogen for vaccine development by the WHO. The glycoprotein (GP) of MARV mediates viral adhesion and invasion of host cells and therefore can be used as an effective target for vaccine development. Moreover, DNA vaccines have unique advantages, such as simple construction processes, low production costs, and few adverse reactions, but their immunogenicity may decrease due to the poor absorption rate of plasmids. Lysosome-associated membrane protein 1 (LAMP1) can direct antigens to lysosomes and endosomes and has great potential for improving the immunogenicity of nucleic acid vaccines. Therefore, we constructed a DNA vaccine based on a codon-optimized MARV GP (ID MF939097.1) fused with LAMP1 and explored the effect of a LAMP targeting strategy on improving the immunogenicity of the MARV DNA vaccine. ELISA, ELISpot, and flow cytometry revealed that the introduction of LAMP1 into the MARV DNA candidate vaccine improved the humoral and cellular immune response, enhanced the secretion of cytokines, and established long-term immune protection. Transcriptome analysis revealed that the LAMP targeting strategy significantly enriched antigen processing and presentation-related pathways, especially the MHC class II-related pathway, in the candidate vaccine. Our study broadens the strategic vision for enhanced DNA vaccine design and provides a promising candidate vaccine for MHF prevention.

## 1. Introduction

Marburg hemorrhagic fever (MHF) is a fatal infectious disease caused by Marburg virus (MARV) infection [1]. There have been 16 sporadic outbreaks of MHF in Africa and other places, with a high case fatality rate of 88% [2]. MARV and Ebola virus (EBOV) belong to the Filoviridae family and are identified as Biosafe level 4 pathogens [3]. The 2014 EBOV outbreak in West Africa became a global public health emergency, causing the public to pay attention to filoviruses [4]. Recently, MHF cases have been reported in Guinea, Ghana, and Tanzania, indicating a potential risk of global outbreaks [5,6,7]. Moreover, MARV has been identified as a priority pathogen for vaccine development by the WHO. Hence, designing and constructing an effective candidate vaccine for MARV is imperative for the prevention and control of MHF. The glycoprotein of MARV is located on the strata externum of the virus and is responsible for regulating viral adhesion and invasion [8,9]. Therefore, GPs can be used as effective targets for MARV vaccine development.

A DNA vaccine is a recombinant eukaryotic expression plasmid containing target protein-coding genes that can express antigen proteins in host cells after immunization and induce effective immune responses. DNA vaccines have unique advantages, including simple construction processes, low production costs, safe operation, easy transportation and storage, and few adverse reactions, making them the preferred strategy for vaccine development [10]. However, the immunogenicity of DNA vaccines in humans and nonhuman primates (NHPs) could decrease due to the poor absorption rate of plasmids [11]. The MARV DNA vaccine RV 247 has been investigated in a phase I trial; 31% of participants produced MARV GP-specific antibodies, and 52% of participants had T-cell responses [12]. An improved injection strategy or sequential immunity could improve the immune effect. Immunization with DNA vaccines through Biojector can stimulate the body to produce greater protection against MARV. Moreover, a stronger CD8^+^ T-cell immune response was induced by sequential immunization with the MARV GP DNA vaccine and the adenovirus carrier vaccine rAd5-GP [13]. However, further exploration of how to improve the immunogenicity of DNA vaccines via this design scheme is needed.

Lysosome-associated membrane protein 1 (LAMP1) is one of the main components of the lysosome membrane; it can direct antigens to lysosomes and endosomes and has great potential for improving the immunogenicity of nucleic acid vaccines [14]. LAMP1 can direct viral antigens to the MHC II compartment to enhance the immune response of CD4^+^ T cells, further activate the humoral response, and promote the activation of CD8^+^ T cells [15,16]. Here, we constructed a DNA vaccine based on a codon-optimized MARV GP and introduced LAMP1 to explore the ability of a LAMP targeting strategy to improve immunogenicity, providing a theoretical basis for MARV DNA vaccine optimization for clinical use.

## 2. Materials and Methods

### 2.1. Recombinant Plasmid

pVAX1-GP_MARV_ was designed based on a codon-optimized MARV GP gene sequence (ID MF939097.1) with a 3× Flag tag and was provided by Sangon Biotech (Shanghai, China) Co., Ltd. The pVAX1-LAMP vector was previously constructed and preserved in our laboratory. pVAX1-LAMP/GP_MARV_ was constructed by inserting the GP gene into pVAX1-LAMP. The size and accuracy of the plasmids were confirmed by agarose gel electrophoresis and Sanger sequencing.

### 2.2. Transfection of HEK293T Cells

HEK293T cells were stored in our laboratory and had been authenticated by STR profiling. The premixed transfection solution was prepared by adding 5 μL of Lipo2000 (Mei5bio, Beijing, China), 1 μg of DNA vector, and fresh medium to each well. After 48 h, the expression of the MARV GP was measured by the corresponding methods.

### 2.3. Quantitative Real-Time PCR

An RNAprep FastPure Kit (TsingKe, Beijing, China) was used to extract RNA. qRT-PCR was conducted using 2× One Step SYBR Master Mix (Vazyme). The PCR conditions were set according to the specifications. The primers used were as follows (5′-3′): MARV-F: TTCATCTGTGGGGTGCCTTC and MARV-R: ATGTCTGTATCCCTGCCCCT-; ACTIN-F: ATCAAGATTGCTCCTCCTGAG and ACTIN-R: CTGCTTGCTGATCCACATCTG. The qPCR experiments were carried out on a thermal cycler (Bio-Rad, Hercules, CA, USA).

### 2.4. Western Blot

The protein samples were obtained using RIPA Lysis Buffer (Beyotime, Shanghai, China) and then centrifuged at 4 °C and 13,200× *g* for 10 min. The supernatant was added to 5× SDS loading buffer, boiled for five minutes, and then subjected to 10% SDS-PAGE (Epizyme, Shanghai, China). An anti-FLAG antibody (Proteintech, Wuhan, China) was used for the detection of antigen proteins. A Fusion FX Imager (Vilber, Lorita, France) was used to obtain images.

### 2.5. Immunofluorescence Analysis

After transfection, the cells were washed, fixed, permeabilized, and blocked. Rabbit anti-FLAG, 488-conjugated goat anti-rabbit antibodies (Proteintech, Wuhan, China), and DAPI (Solarbio, Beijing, China) were used. Finally, a confocal microscope (FV3000, Olympus, Tokyo, Japan) was used to obtain images.

### 2.6. Prokaryotic Expression of MARV GP

To obtain the MARV GP, we constructed the pET28a-GP_MARV_ vector containing a 6× His tag. Isopropyl β-D-1-thiogalactopyranoside (0.8 mM) was added to stimulate protein expression, and a His-tag Protein Purification Kit (Beyotime) was used to purify the GP protein.

### 2.7. Animals and Immunization

Female BALB/c mice (6–8 weeks) were obtained from the Laboratory Animal Centre of our university (groups = 4, n = 14). Mice received the DNA vector (30 μg) or isopycnic PBS by intramuscular injection using a 1 mL sterile syringe at weeks 0, 4, and 24. Serum samples were collected by tail vein bleeding.

### 2.8. Enzyme-Linked Immunosorbent Assay (ELISA)

The purified MARV GP (10 μg/mL) was used for coating the ELISA plate (Costar, Washington, DC, USA) overnight at 4 °C. The next morning, the plates were washed and blocked. Then, the mouse sera were serially diluted (1:100 to 1:500,000), added to the wells, and preserved at 37 °C for 1 h. After washing, HRP-conjugated goat anti-mouse IgG (CST, Hong Kong, China) was used as a secondary antibody. The TMB substrate and ELISA stop solution (Solarbio) were used for chromogenic reactions. The absorbance was obtained by a microplate reader (TECAN, Männedorf, Switzerland).

### 2.9. Serum Neutralization Test

The mouse sera were serially diluted (1:10 to 1:160) and mixed with 100-fold TCID_50_ of the rVSV-ΔGP_EBOV_ pseudovirus (previously stored in our laboratory) [16,17]. After incubation at 37 °C for 1 h, the mixed liquid was added to 96-well plates with Vero E6 cells. After 2 h, the supernatant was discarded, and fresh medium was added. After incubation for 36 h, the infection of the cells was observed, and the 50% protective dose (PD_50_) was calculated by using Karber’s method.

### 2.10. Synthesis of GP Peptides

The dominant epitopes of the MARV GP were predicted by using IEDB. We also performed cluster analysis, conservative analysis, and molecular docking of the dominant epitopes, as previously described [18]. The peptides used for this study contained 15 kinds of 9-mer peptides, 4 sets of 15-mer peptides, and 1 each of 16/18/20-mer peptides. All of the peptides were synthesized by Apeptide Biotech Ltd.(Shanghai, China)

### 2.11. Enzyme-Linked Immunospot Assay (ELISpot)

Mouse IFN-γ and IL-4 ELISpot Kits (BD Pharmingen) were used to perform ELISpot experiments following the manufacturer’s instructions. After each booster immunization, mouse spleens were ground in 1640 medium. After erythrocyte lysis, the spleen cells were added to the plates (1 × 10^6^ cells/well). Peptides were used for stimulation (30 μg/mL). Concanavalin A was added in positive control wells (2 μg/mL, Sigma, Milwaukee, United States). An HRP 3-amino-9-ethylcarbazole peroxidase substrate kit (Solarbio, Beijing, China) was used for chromogenic reactions. The spots were counted and analyzed by an ELISpot plate reader (CTL, Cleveland, OH, USA).

### 2.12. Flow Cytometry

For effective memory T (Tem) cell detection, cells were stimulated with mixed 15/16/18/20-mer peptides (2 μg/mL per peptide). For cytokine evaluation, a cell stimulation cocktail (Invitrogen, Carlsbad, CA, USA) was used. Both were incubated at 37 °C for 4 h. Cytofix/Cytoperm solution (BD) was used for fixation and permeabilization. The following antibodies (BioLegend, San Diego, CA, USA) were used: CD3-APC-Cyanine7, CD4/CD8-FITC, CD44-PE, and CD62L-APC for Tem cell detection; and CD3-FITC, CD4-PE, CD8-Pacific Blue, and IL-2/IL-4/IFN-γ-APC for cytokine evaluation. The flow cytometry assays were performed with a NovoCyte Flow Cytometer (ACEA Biosciences, San Diego, CA, USA), as previously described [19].

### 2.13. Transcriptome Analysis

Mouse splenocytes were collected and stimulated with peptides (30 μg/mL) for 24 h at 37 °C and then preserved in TRIzol reagent (Vazyme, Nanjing, China) at −80 °C. Transcriptome sequencing was performed by Seqhealth Ltd. (Wuhan, China).

### 2.14. Animal Behavior Analysis

After long-term immunization, mouse behavior within 24 h was evaluated by the VB-AIH multichannel behavior automatic analysis system (Vanbi, Shanghai, China).

### 2.15. Hematoxylin and Eosin (H&E) Staining

After long-term immunization, the main organs of the mice were collected, fixed, and stained with hematoxylin and eosin (H&E, Hong Kong, China). The images were obtained by using OLYMPUS VS200 (Tokyo, Japan).

### 2.16. Statistical Analysis

GraphPad Prism 8.0 software was used to analyze data. Statistical significance among different groups was evaluated using unpaired t-tests (* *p* < 0.05, ** *p* < 0.01, *** *p* < 0.001, **** *p* < 0.0001).

## 3. Results

### 3.1. Construction and Verification of Plasmids

**Figure 1:**
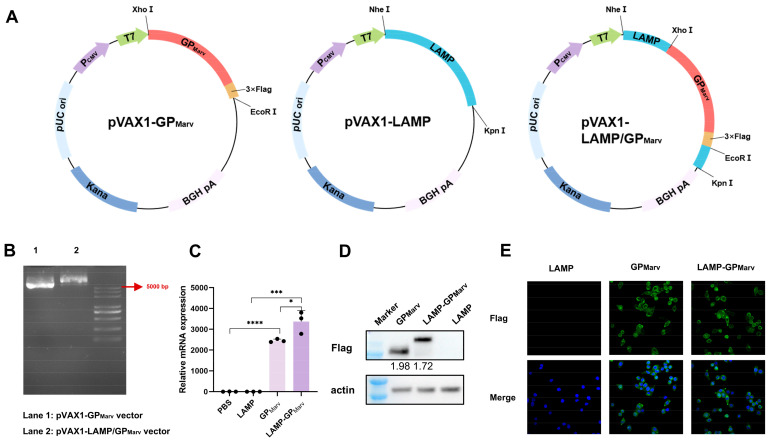
The construction and verification of the plasmids. (**A**) The codon-optimized MARV GP gene sequence was used to construct the pVAX1-GP_MARV_ plasmids. pVAX1-LAMP/GP_MARV_ was constructed by inserting the GP gene into the pVAX1-LAMP vector. (**B**) The sizes of the recombinant plasmids were verified by agarose gel electrophoresis. (**C**) The relative mRNA expression of the MARV GP was detected by qPCR. (**D**) The GP in the transfected cells was verified by Western blotting. (**E**) Immunofluorescence images of the MARV GP in transfected cells (40×) (* *p* < 0.05, *** *p* < 0.001, **** *p* < 0.0001).

Codon optimization of the MARV GP gene sequence (ID MF939097.1) was performed, and a 3× Flag tag was subsequently added to the sequence, which was then used to construct the pVAX1-GP_MARV_ plasmid. pVAX1-LAMP/GP_MARV_ was constructed by inserting the GP gene into the pVAX1-LAMP vector (Figure 1A). The sizes of the recombinant plasmids were verified by agarose gel electrophoresis (Figure 1B). The expression of the MARV GP was detected after transfection, and the qPCR and Western blot results showed that both vectors could successfully express the MARV GP (Figure 1C,D). Immunofluorescence images showed that the distribution of the MARV GP was mainly in the cytoplasm regardless of the presence of LAMP1 (Figure 1E).

### 3.2. pVAX1-LAMP/GP_MARV_ Induced a Stronger Humoral Immune Response

**Figure 2:**
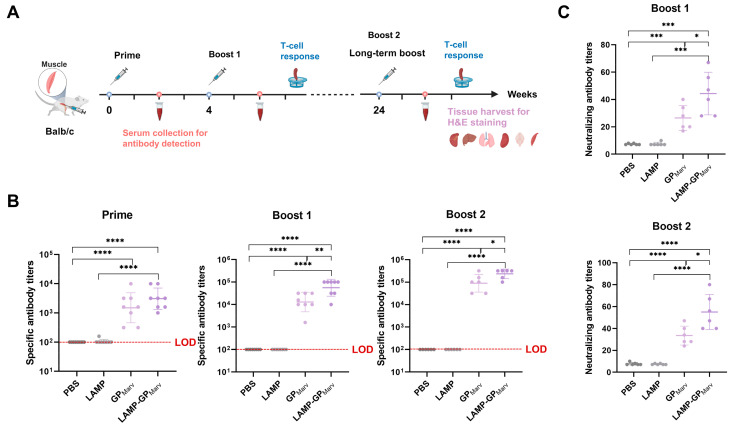
The evaluation of the humoral immune response induced by the candidate vaccines. (**A**) The schedule of immunization, sample collection, and experiments. (**B**) The MARV GP-specific antibody titers were detected by ELISA after each immunization. (**C**) The cross-neutralizing antibody titers for EBOV were detected after each booster immunization (* *p* < 0.05, ** *p* < 0.01, *** *p* < 0.001, **** *p* < 0.0001).

The immunization schedule is shown in Figure 2A. A total of three immunizations were performed in BALB/c mice by gastrocnemius injection. The MARV GP-specific antibody titers were detected by ELISA (Figure 2B). After the first dose, both of the experimental groups (GP and LAMP-GP) produced specific antibodies compared with the control groups (PBS and LAMP). Moreover, the specific antibody titers were significantly increased after booster immunization, suggesting that long-acting protective immunity was successfully established. Notably, the level of antibodies was higher in the mice that received LAMP-GP than in the mice immunized with the GP after booster immunization, indicating that pVAX1-LAMP/GP_MARV_ induced a stronger humoral immune response. In addition, cross-neutralizing antibody titers for EBOV were detected, and both of the experimental groups produced neutralizing antibodies (Figure 2C). The mice that received pVAX1-LAMP/GP_MARV_ exhibited greater levels of neutralizing antibodies than those that received pVAX1-GP_MARV_, suggesting that the introduction of LAMP1 could enhance the humoral response.

### 3.3. The LAMP Targeting Strategy Enhanced the Secretion of IFN-γ and IL-4

**Figure 3:**
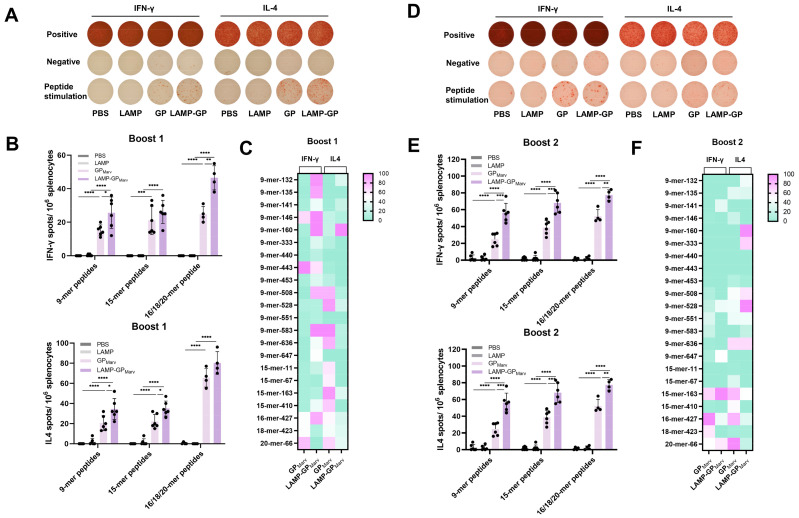
The production of IFN-γ and IL-4 was evaluated by ELISpot after each booster immunization. (**A**) Representative images of IFN-γ and IL-4 spots after booster immunization. (**B**) The levels of cytokines induced by the candidate vaccines were detected under stimulation with different peptide pools. (**C**) The stimulation effect of a single peptide is also shown as a heatmap. (**D**) Representative images of spots after long-term booster immunization. (**E**) The secretion of cytokines was further detected in mice that received long-term booster immunization. (**F**) The stimulatory effect of a single peptide after long-term booster immunization (* *p* < 0.05, ** *p* < 0.01, *** *p* < 0.001, **** *p* < 0.0001).

The secretion of cytokines partly reflected the T-cell response. An ELISpot assay was conducted to evaluate the levels of IFN-γ and IL-4 after booster immunization. After the first booster immunization, both of the experimental groups produced IFN-γ and IL-4 spots, and the representative images are listed in Figure 3A. Compared with PBS or LAMP, the mice that received the GP and LAMP-GP produced more IFN-γ and IL-4, and there were more IL-4 spots than IFN-γ spots (Figure 3B). After comparing the spots under stimulation with different peptide pools, we found that 16/18/20-mer peptides had a better effect on stimulation. Moreover, the stimulatory effect of a single peptide was also evaluated, and the 16-mer peptide led to increased production of IFN-γ and IL-4 in both experimental groups (Figure 3C).

After long-term booster immunization, the IFN-γ spots appeared larger than those after the first booster immunization (Figure 3D). Moreover, the number of IFN-γ spots was also increased, indicating that a long-term Th1-type cell response was established. Notably, the LAMP-GP group produced markedly more IFN-γ and IL-4 than the GP group under stimulation with each peptide pool, suggesting that the LAMP targeting strategy could improve the cell response in immunized mice (Figure 3E). In addition, the 15-mer-163 and 16-mer peptides exhibited better stimulatory effects (Figure 3F).

### 3.4. Flow Cytometry Revealed That LAMP1 Can Enhance the T-Cell Response

**Figure 4:**
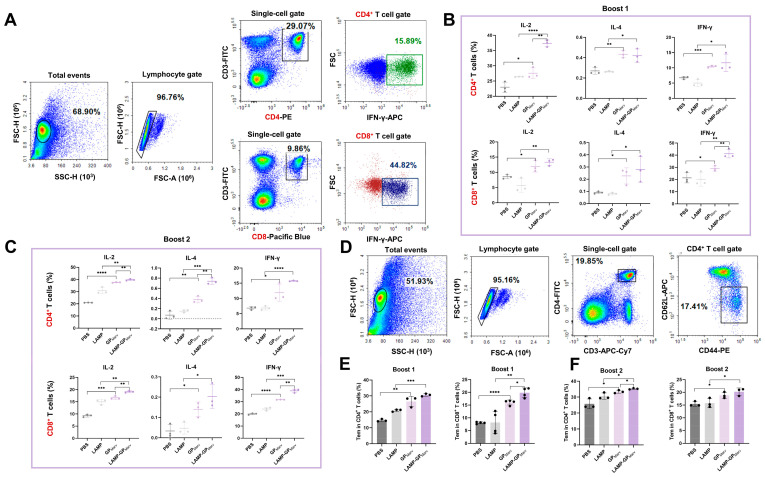
The T-cell response was evaluated in mice that received the candidate vaccines by flow cytometry. (**A**) The gating graphs for CD4^+^ and CD8_+_ T cells (using IFN-γ as an example). (**B,C**) The secretion of IL-2, IL-4, and IFN-γ by CD4^+^ and CD8_+_ T cells was detected after each booster immunization. (**D**) Gating graphs for the CD4^+^ Tem cells. (**E,F**) MARV GP-specific CD4^+^ and CD8^+^ Tem cells were observed after each booster immunization (* *p* < 0.05, ** *p* < 0.01, *** *p* < 0.001, **** *p* < 0.0001).

To better elucidate the cell response induced by the candidate vaccines, the secretion of cytokines by CD4^+^ and CD8^+^ T cells was further detected by flow cytometry. A four-color fluorescence labeling antibody scheme was applied, and the gating graphs for CD4^+^ and CD8^+^ T cells are presented in Figure 4A (using IFN-γ as an example). After the first booster injection, the experimental groups exhibited greater secretion of cytokines in CD4^+^ and CD8^+^ T cells than the control groups did, and the LAMP-GP group produced more IL-2 in CD4^+^ T cells and more IFN-γ in CD8^+^ T cells than the GP group did, indicating that both of the candidate vaccines could effectively activate the cellular immune response, and the LAMP strategy showed advantages in some respects (Figure 4B). After long-term booster immunization, the cytokine secretion of both experimental groups increased to different degrees, and the LAMP-GP group showed advantages in the production of IL-2/IL-4 in CD4^+^ T cells and IL-2/IFN-γ in CD8^+^ T cells, which further validates the potential benefits of the LAMP targeting strategy (Figure 4C).

Given that effective memory T (Tem) cells can reflect long-term immune protection, the levels of CD4^+^ and CD8^+^ Tem cells were also detected. The gating graphs for CD4^+^ Tem cells are shown in Figure 4D, which were also applied to the detection of CD8^+^ Tem cells. After the first booster immunization, a remarkable increase in the level of MARV GP-specific CD4^+^ and CD8^+^ Tem cells was found in both experimental groups, and the LAMP-GP group exhibited more CD8^+^ Tem cells than the GP group did (Figure 4E). Moreover, the CD4^+^ Tem cells increased after long-term booster immunization, and the levels of CD4^+^ Tem cells in the LAMP-GP group were greater than those in the GP group (Figure 4F). Collectively, these results indicate that a long-term cellular immune response was established and that a LAMP targeting strategy could enhance the T-cell response induced by candidate vaccines.

### 3.5. Transcriptome Analysis of Immune Response-Related Pathways

**Figure 5:**
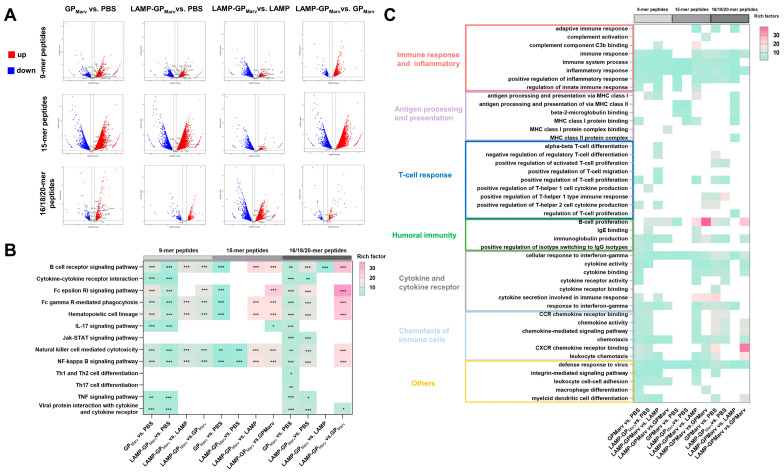
Transcriptome analysis of immune response-related pathways. (**A**) Volcano plots showing the upregulated and downregulated genes in different groups under stimulation with different peptide pools. (**B**) KEGG enrichment analysis revealed that both of the candidate vaccines activated immune response-related pathways, especially the B-cell receptor signaling pathway and NK cell-mediated cytotoxicity pathways. (**C**) Both of the candidate vaccines affected the overall immune response and inflammation-related pathways (* *p* < 0.05, ** *p* < 0.01, *** *p* < 0.001).

RNA-seq and transcriptome analysis were performed after booster immunization to reveal the underlying mechanism of the immune response stimulated by the candidate vaccines. Volcano plots show the upregulated and downregulated genes in different groups under stimulation with different peptide pools. The genes encoding a number of chemokines and chemokine receptors (CCL3/4/5/67 and CXCL2/3/5/9/10) and cytokines (IL-2/4, IFNG, and GZMB) were upregulated in the experimental groups, suggesting that immune cells were effectively activated by the candidate vaccines (Figure 5A). KEGG enrichment analysis revealed that both of the candidate vaccines activated immune response-related pathways, especially the B-cell receptor signaling pathway and NK cell-mediated cytotoxicity pathways (Figure 5B). GO enrichment analysis revealed that both of the candidate vaccines affected the overall immune response and inflammation-related pathways (Figure 5C). Notably, under stimulation with 16/18/20-mer peptides, the antigen processing and presentation-related pathways were significantly enhanced and activated in the LAMP-GP group, especially the MHC class II-related pathways, which further validated the advantages of the LAMP targeting strategy in antigen processing and presentation. Additionally, both of the candidate vaccines upregulated the expression of T-cell response and humoral immunity-related pathway-related genes, indicating that the candidate vaccines effectively induced immune effects.

### 3.6. Preliminary Safety Assessment of Candidate Vaccines

**Figure 6:**
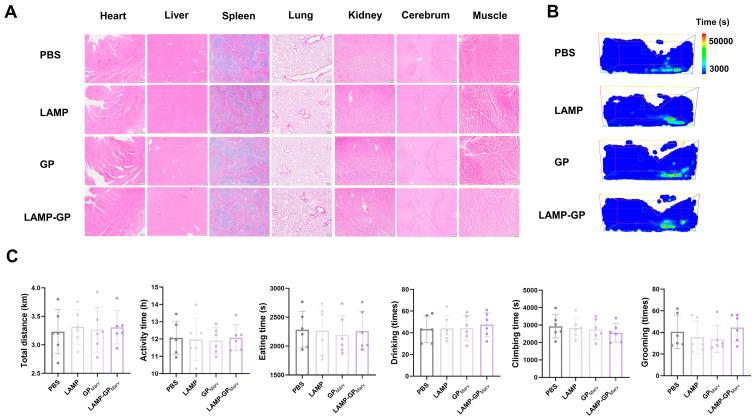
Preliminary safety assessment of candidate vaccines. (**A**) H&E staining was performed on the main organs of mice after long-term booster immunization. (**B**) Representative heatmap of animal behavior recorded by an automatic analysis system within 24 h. (**C**) The activity time and distance, eating and drinking, grooming, and climbing data were collected and analyzed.

The main organs of the mice were collected after long-term booster immunization, and H&E staining images showed that the candidate vaccines did not lead to pathological alterations (Figure 6A). Additionally, the behavior of the animals in the different groups was evaluated, and a heatmap is shown in Figure 6B. The candidate vaccines did not result in any changes in the diet or behavioral habits of the mice (Figure 6C).

## 4. Discussion

The initial symptoms of patients infected with MARV are fever, diarrhea, vomiting, and skin symptoms, and more severe symptoms can occur after an incubation period of approximately two weeks, including hypotension, shock, and multiple organ failure [20]. MHF has a high fatality rate, and few clinical treatments or specific drugs can be used [21]. Therefore, vaccine development is highly important for MHF prevention and control. In this study, we constructed a novel MARV DNA vaccine fused with LAMP1 and demonstrated that the LAMP targeting strategy significantly improved the immunogenicity of the candidate vaccine and improved humoral and cellular immune effects in immunized mice, providing a potentially effective candidate vaccine for preventing and controlling MHF epidemics.

Considering the characteristics of lysosomal membrane proteins, a LAMP targeting strategy has been explored for a variety of vaccines [22,23,24,25]. Su et al. used the lysosomal targeting properties of LAMP1 to construct a CryJ-LAMP vaccine, which can induce mice to produce high levels of IFN-γ and has the potential to treat allergies induced by Japanese bacteria by improving the Th1/Th2 status of the body [26]. Hollý et al. inserted the antigen sequence into the transmembrane domain and cytoplasmic domain tail of LAMP2, constructed the influenza vaccine pEx 4M2e-LAMP-2a, and demonstrated that the introduction of LAMP2 increased the level of specific antibodies 16-fold [27]. In addition, Teixeira et al. constructed the HIV DNA vaccine LAMP-1/p55Gag, which can induce the maturation of early T follicular helper cells and the formation of germinal center sites, indicating that the LAMP1 strategy may even benefit newborns [28]. Our laboratory previously constructed a LAMP1-fused DNA vaccine against the GN and GC of Hantavirus, which can induce both virus-specific and neutralizing antibodies and establish long-term immune protection [15,29]. The advantages of LAMP1 were further validated in the EBOV DNA vaccine, in which the immunized mice exhibited better humoral and cellular immune responses and produced more neutralizing antibodies [16]. This study first constructed a MARV vaccine containing LAMP1 and showed that the LAMP targeting strategy could more effectively activate humoral and cellular immune responses specific to the MARV GP and establish a long-lasting immune response, which further confirmed the effectiveness of the LAMP targeting strategy in the development of viral vaccines. In future studies, we will attempt to explore and compare other targeting strategies or adjuvants to see if they offer better or complementary effects to LAMP1 in enhancing the vaccine’s immunogenicity.

The activation of humoral immunity is an important index for evaluating the efficacy of vaccines. Riemenschneider et al. constructed a MARV GP DNA vaccine and injected 2.5 μg of DNA into guinea pigs via a gene gun at weeks 0, 4, and 8. Finally, a specific antibody titer of 10^3^ was detected via ELISA [30]. In contrast, the dose used in our study was 30 μg, and the immunization schedule was at weeks 0, 4, and 24. The DNA candidate vaccine without LAMP1 produced MARV GP-specific antibody titers of 9 × 10^4^, suggesting that under this dose and immunization schedule, better long-lasting humoral immunity could be obtained even without the use of a gene gun. Moreover, Geisbert et al. constructed the MARV DNA vaccine pGP and immunized monkeys with 4 mg of DNA; the antibody titer was approximately 5 × 10^3^ after three immunizations, and after enhanced immunization with rAd5, the average titer increased to 10^5^ [13]. Our vaccine dose was 30 µg per mouse, and the antibody titer obtained by LAMP-GP exceeded 10^5^ after three immunizations, reflecting the advantages of the LAMP targeting strategy. In addition, Bukreyev et al. constructed an mRNA vaccine for MARV, and the immunized guinea pigs obtained a specific antibody titer of 1:8192 after three immunizations [31], while our LAMP-GP DNA vaccine obtained a specific antibody titer of approximately 1:100,000, indicating that the DNA vaccine may have advantages over the mRNA vaccine in humoral immunity. Overall, our LAMP-GP candidate vaccine effectively activated humoral immune responses and established long-lasting humoral immune protection.

Both CD4^+^ and CD8^+^ T cells are essential for the cellular immune response. CD4^+^ T cells can promote the production of a large number of cytokines, including IFN-γ, TNF-α, IL-2, and IL-12, by CD8^+^ T cells. These cytokines can enhance CD8^+^ T-cell toxicity and further maintain the survival of CD8^+^ T cells [32,33]. The ELISPOT assay is capable of detecting multiple cytokines or cytotoxic substances at the single-cell level [34]. Therefore, we used ELISPOT to evaluate IFN-γ and IL-4 levels in immunized mice, and the results showed that the LAMP-GP group produced higher levels of IFN-γ and IL-4, indicating that the addition of LAMP1 to the candidate DNA vaccine enabled more T cells to be activated. Moreover, flow cytometry further verified that the LAMP targeting strategy could enhance the secretion of IL-2, IL-4, and IFN-γ by CD4^+^ and CD8^+^ T cells. In terms of long-term immune memory, the LAMP-GP group also exhibited increased CD4^+^ and CD8^+^ Tem cell levels after booster immunization, indicating the benefits of the LAMP targeting strategy in establishing a long-term cellular immune response. In addition, transcriptome analysis further verified the advantages of the LAMP strategy in antigen processing and presentation. These results showed that LAMP1 could enhance the T-cell immune response induced by the candidate vaccine through enrichment of the antigen presentation pathway.

This study has several limitations. First, although the injection doses of the GP and LAMP-GP DNA were both 30 μg, the antigen copy number in the LAMP-DNA group decreased due to the introduction of LAMP1. Future studies should be proposed to further optimize the LAMP1 sequence to exert its targeting function with a minimum length. Second, given that viruses can escape immune surveillance through the glycosylation of GPs, galactosylation could be considered in vaccine design to activate immune protection more effectively [35]. Third, although preliminary safety has been assessed by H&E and animal behavior evaluation, the efficiency and safety of the current candidate vaccine need to be verified in NHPs and with virus challenge experiments in future studies.

## 5. Conclusions

In summary, the present study is the first to apply a LAMP targeting strategy to construct a MARV DNA vaccine and demonstrates that the introduction of LAMP1 as a candidate vaccine could improve humoral and cellular immune responses, enhance cytokine secretion, and establish long-term immune protection. Our study broadens the strategic vision for enhanced DNA vaccine design and provides a promising candidate vaccine for MHF prevention.

## Data Availability

The datasets generated and analyzed during the current study are available from the corresponding author upon reasonable request.
